# Choice of measure matters: A study of the relationship between socioeconomic status and psychosocial resources in a middle-aged normal population

**DOI:** 10.1371/journal.pone.0178929

**Published:** 2017-08-23

**Authors:** Karin Festin, Kristin Thomas, Joakim Ekberg, Margareta Kristenson

**Affiliations:** 1 Division of Community Medicine, Department of Medical and Health Sciences, Faculty of Medicine and Health, Linköping University, Linköping, Sweden; 2 Unit for Health Analysis, Centre for Healthcare Development, Region Östergötland, Linköping, Sweden; CUNY, UNITED STATES

## Abstract

Psychosocial resources may serve as an important link to explain socioeconomic differences in health. Earlier studies have demonstrated that education, income and occupational status cannot be used interchangeably as indicators of a hypothetical latent social dimension. In the same manner, it is important to disentangle the effect of measuring different constructs of psychosocial resources. The aim of this study was therefore to analyse if associations between socioeconomic status (SES) and psychosocial resources differ depending on the measures used. A cross-sectional population-based study of a random sample (n = 1007) of middle-aged individuals (45–69 years old, 50% women) in Sweden was performed using questionnaire and register data. SES was measured as education, occupation, household income and self-rated economy. Psychosocial resources were measured as social integration, social support, mastery, self-esteem, sense of coherence (SOC) and trust. Logistic regression models were applied to analyse the relationships controlling for the effects of possible confounders. The measures of SES were low or moderately correlated to each other as were the measures of psychosocial resources. After controlling for age, sex, country of birth and employment status, household income and self-rated economy were associated with all six psychosocial resources; occupation was associated with three (social integration, self-esteem and trust) and education with two (social integration and self-esteem). Social integration and self-esteem showed a significant and graded relationship with all SES measures; trust was associated with all SES measures except education, whereas SOC and mastery were only associated with household income and self-rated economy. After controlling for other SES measures, no associations with psychosocial resources remained for education or occupation. In conclusion, associations between SES and psychosocial resources did differ depending on the measures used. The findings illustrate the importance of the choice of measure when investigating SES as well as psychosocial resources.

## Introduction

The relationship between socioeconomic status (SES) and health is well known. Numerous studies have shown strong and consistent gradients whereby individuals with low SES have a greater risk of poor health compared with individuals with higher SES [1–5]. Inequalities have been found for most health outcomes and have shown to be particularly pronounced for coronary heart disease (CHD), with an almost twofold difference in the incidence of CHD between high and low education groups [1]. In studies of inequalities in health SES has, typically been investigated by level of education, occupation or income [1–6].

In the search for explanations for SES differences in health, it has been demonstrated that unhealthy lifestyles, which are well-known health risk factors and more prevalent in low SES groups, can only partly explain SES health disparities [7]. A recent review on possible causes of persisting SES health disparities argued that targeting personal, psychosocial and cultural determinants may be necessary to achieve a substantial reduction in health inequalities [8].

The evidence for a psychosocial impact on CHD, independent of unhealthy lifestyle and other risk factors, is large and increasing [9–14]. Psychosocial risk factors found to be related to an increased risk of CHD include psychosocial work environment, low social support, perceived stress, anxiety and depression [9–14]. Similar to lifestyle, psychosocial risk factors are also distributed unevenly across SES groups, and have also, partly, explained SES differences in health [6,15]. However, a recent review of the pathways between SES and health concluded that psychosocial resources may be an even more promising pathway [6].

Psychosocial resources in the social environment, in terms of both social integration and emotional support have been found to significantly influence health [6,12]. Among psychological resources, mastery, sense of coherence (SOC) and self-esteem, have been shown to have protective effects on all-cause mortality, cardiovascular disease and cancer [16–19]. Our group published the first population-based prospective study showing the protective effect of mastery and self-esteem on first-incident myocardial infarction [16]. In addition to these more established psychosocial resources, social trust has been found to be associated with self-rated health and somatic outcome [20–22]. Similar to psychosocial risk factors, people with low SES also tend to have lower levels of psychosocial resources in terms of both a supportive social environment [23] and psychological resources [6,15], thus having limited resilience to deal with stressors such as material deprivation. Differences in resilience could therefore explain the emerging picture of increased general vulnerability in low SES groups and serve as one important link to explain differences in SES in CHD [15,24].

In studies of health inequalities, references are often made to one indicator of SES to support results from another indicator of SES [25]. This practice of using different indices of SES interchangeably has been questioned on several grounds. This leads to theoretical confusion because it is unlikely that education, income and occupational status are related to the same underlying dimension. Different measures of SES signify access to different forms of resources and prestige where each indicator represents different aspects. Education gives access to knowledge and competency resulting in e.g. increased ability to gain work, flexibility on the labour market and increased health literacy. Income-based indicators encompass access to material and immaterial resources for health e.g. housing, clothing and food, and resources to control one’s circumstances but also denotes a level of prestige. Occupational can promote perceptions of professional identity and community, and is an important marker for working conditions, both physical and psychosocial [25,26]. Indeed, studies have shown that the effects of the above-described SES measures differ depending on health outcome. Therefore, although correlated, education, income, and occupational status cannot be used interchangeably as indicators of a hypothetical latent social dimension [25].

As for measures of constructs, the psychosocial resources described above also exhibit different characteristics. Measures of social environment include quantitative measures of social integration and qualitative measures of emotional support and attachment [27]. Although the constructs of mastery and self-esteem are closely related, they present distinct domains. Mastery, or coping ability, captures feelings of confidence and self-reliance, including perceptions that life to some extent is manageable, whereas self-esteem denotes the perception of basic self-worth [28,29]. The construct of SOC is a three-dimensional construct describing to what extent life is perceived as comprehensible, manageable, and meaningful [30]. Trust has been defined as a characteristic belief that the sincerity, good will, or truthfulness of others can generally be relied upon [31]. Thus, psychosocial resources do represent different construct and could subsequently influence health inequalities in different ways. If we aim to understand the role of psychosocial resources in health inequalities, it is therefore important to disentangle the respective contributions of different measures of both SES and psychosocial resources. The present, cross-sectional analysis, which includes a comprehensive set of these measures, is a preparatory step within a larger prospective study within the above aim.

### Study aim

To analyse if associations between SES and psychosocial resources differ depending on the measures used.

## Methods

### Subjects and procedures

The present study is a part of the Life conditions, Stress and Health (LSH) research program, which aims to prospectively investigate the role of psychosocial factors in SES differences in the incidence of CHD. Data collection was conducted in collaboration with ten primary care centres in the County of Östergötland in Sweden from October 2003 to May 2004.

A random population-based sample of individuals (between 45 and 69 years of age) were invited consecutively until a study population size of 1007 was reached, evenly distributed by age and sex (505 women and 502 men). Representatives at the primary care centres obtained written informed consent from all participants. The response rate was 62.5%. Exclusion criteria were serious physical conditions, e.g. terminal cancer, or psychiatric illnesses that would interfere with practical procedures. Data on SES (self-rated economy, occupation and education), psychosocial resources and background information were collected through questionnaires. Data on household income were collected from register data from Statistics Sweden.

### Measures

#### Socioeconomic status

SES was measured as education, occupational status, and income. Educational status was based on self-reported data and categorized into four groups according to the highest completed level: primary school, secondary school (2 years or 3–4 years) and university. Occupational status was also based on self-reported data and concerned occupation during the major part of life. The data was classified using the Swedish socioeconomic classification system (SEI) [32] whereby three categories were used: manual workers, non-manual employees and self-employed (including farmers). For income, two measures were used: household income and self-rated economy. Household income was measured using register data from Statistics Sweden on disposable household income in 2003. Disposable household income includes income from employment and capital and the net value of positive transfers (allowances of various types) and negative transfers (taxes and contributions). Household income was categorized into quartiles. Self-rated economy was measured using one item: “How would you rate your economy” with five possible answers; the three lower categories (“very bad”, “quite bad”, and “neither good nor bad”) were merged into one (“not good”) and the other two remained as “good” and “very good”.

#### Psychosocial resources

To measure the social environment, two components from the abbreviated instrument Interview Schedule for Social Interaction (ISSI) were used, both consisting of six items. The first component, Availability of Social Integration (AVSI), investigates the availability of social contacts, e.g. friends, work associates and acquaintances. The second component, Availability of Attachment (AVAT), investigates emotional support, i.e. the availability of affectional close relationships [27].

Psychological resources were investigated using four instruments. Mastery was measured using an instrument including seven items that investigate the extent to which one regards one’s life as being under one’s control in terms of confidence and self-reliance [28]. Self-esteem was measured using a ten-item instrument investigating a global dimension of self-worth and self-esteem in comparison with other people’s competences [29]. SOC was measured using the 13-item version of the instrument by Antonovsky [33]. SOC reflects the extent to which one feels one’s own life is comprehensible, manageable, and meaningful. Finally, trust was measured with a single item “Do you feel that you can trust people?” [31].

#### Background information: Possible confounders

Age and sex were adjusted for throughout the analyses. In addition, employment status and country of birth were also controlled for as they have been found to influence the SES-health relationship [34,35]. Country of birth and employment status were self-reported by a questionnaire. Country of birth was dichotomized into born in a Nordic country or born in a non-Nordic country. Employment status was categorized into three groups: those who were employed and worked at least part time, full-time unemployed and full-time pensioners (due to high age or disability).

### Statistical data analysis

Background and socioeconomic characteristics were described as number and percentage, mean or median, standard deviation or interquartile range. The number of self-employed individuals was low. Thus, in the analyses of relationships to occupational status, only the categories manual workers and non-manual employees were considered.

For all psychosocial instruments scale sores were summarized, and for all, expect trust, internal consistency was calculated with Cronbach’s alpha. Each scale was divided into quartiles and thereafter dichotomized into quartiles 1–3 “low scores” versus quartile 4 (highest scale scores) “high scores”. This procedure was true for all instruments except social support and trust, which because of a ceiling effect had to be dichotomized into quartile 1 versus quartiles 2–4. Spearman’s rank correlation coefficient was used to determine the relationship between measures of SES and psychosocial resources. The prevalence of participants with high scale scores of psychosocial resources over SES levels were graphically illustrated for each of the four SES measures. In the statistical analyses the lowest category within each SES measure (e.g. primary school) was used as reference. The Bonferroni method was applied for these comparisons.

Three steps of logistic regression models were used. In the first step, logistic regression analyses were used to investigate the relationship between each of the four SES measurements and the six psychosocial resources as well as with employment status and country of birth. Those models were adjusted for age and sex. In the second step, the logistic regression models with SES and psychosocial resources were also adjusted for employment status and country of birth. In the third step, all models in the second step were also mutually adjusted for other SES measurements, to assess the independent relationship of each SES measure [25]. This was done firstly one by one and thereafter for all other SES measures. Because of the risk for collinearity between self-rated economy and psychosocial resources, both being self-reported measures, we decided not to adjust for self-rated economy in this stage. However, the models with self-rated economy were adjusted for education, occupational status and household income. A p-value for trend was calculated for the SES measures education, household income and self-rated income. For nominal variables (occupation, country of birth and employment status), the p-value indicates the Wald statistic. Odds ratios with 95% confidence intervals were calculated. Possible interaction of sex on the relationship between SES and psychosocial factors was investigated by including an interaction term in the regression models. A two sided probability value of p≤0.05 was considered to be statistically significant. Analyses were performed in SPSS, release 24.

### Ethical considerations

The study was approved by the Regional Ethics Review Board of Linköping University, Linköping, Sweden (02–0324).

## Results

Table 1 presents background and socioeconomic characteristics of the population in terms of age, sex, education, occupation, disposable household income, self-rated economy, country of birth and employment status. The participants were on average 57 years old and 50% of them were women. Almost two thirds were employed and about half were, or had been, non-manual employees. More than one third had 9 years of education (primary school). Median household income was €33,500 and 29% of the participants rated their economy as very good. The study sample was representative of the population in terms of educational attainment, immigrant status, and employment status.

**Table 1:** Background and socioeconomic characteristics (n = 1007).

Variable	Category	n	%	Mean or median	SD or IQR
**Age**		1007	100	57.0	7.1
**Sex**					
	Female	502	49.9		
	Male	505	50.1		
**Education**					
	Primary school	352	35.6		
	Secondary school (2 years)	294	29.8		
	Secondary school (3–4 years)	133	13.5		
	University	209	21.2		
**Occupation**^1^					
	Manual workers	418	43.0		
	Non-manual employees	502	51.6		
	Self-employed and farmers	52	5.4		
**Household income**^2^		1007	100	33,646	21,904; 45,033
	Q1	251	24.9	16,443	13,068; 19,445
	Q2	252	25.0	27,054	24,701; 30,478
	Q3	252	25.0	38,475	36,378; 41,445
	Q4	252	25	54,839	49,068; 64,055
**Self-rated economy**					
	Not good	223	22.1		
	Good	467	48.2		
	Very good	278	28.7		
**Country of birth**					
	Nordic countries	945	94.6		
	Other	54	5.4		
**Employment status**					
	Working	614	61.0		
	Unemployed	111	11.0		
	Pensioner (disability or age)	282	28.0		

^1^Swedish socioeconomic classification (SEI).

^2^2003 (€).

Characteristics of the psychosocial measurements are shown in Table 2. Internal consistency in terms of Cronbach’s alpha ranged from 0.72 to 0.88. The range of scale scores in the study population covered the main part of the possible range in the instruments.

**Table 2:** Characteristics of psychosocial resources in the study population.

Psychosocial measure	Number of items	Cronbach’s alpha	Range in instrument	Range in study population	Mean (SD)	Median (IQR)
Social integration^1^ (n = 957)	6	0.88	0–36	6–36	20.5 (5.9)	20 (16; 24)
Social support^2^ (n = 959)	6	0.77	0–6	0–6	5.5 (1.1)	6 (5; 6)
Mastery (n = 943)	7	0.76	7–28	7–28	22.6 (3.4)	23 (20; 25)
Self-esteem (n = 942)	10	0.86	10–40	15–40	32.2 (4.8)	33 (30; 36)
SOC^3^ (n = 957)	13	0.82	13–91	32–91	68.7 (10.4)	70 (62; 77)
Trust (n = 930)	1	–	1–5	1–5	4.0 (0.6)	4 (4; 4)

^1^AVSI.

^2^AVAT.

^3^Sense of coherence.

As seen in Table 3, the correlation coefficients between the four SES measures were generally weak or moderate, ranging from 0.14 (education and self-rated economy) to 0.44 (education and occupational status). Overall, measures of psychosocial resources showed stronger associations to one another, however with large variation, and the correlation ranged from 0.12 (trust and social support) to 0.68 (mastery and self-esteem). Measures of self-rated economy and household income showed stronger correlations with the psychosocial measurements compared with occupational status and education.

**Table 3:** Intercorrelation matrix for measures of socioeconomic status and psychosocial resources.

	Education	Occupational status^1^	Household income	Self-rated economy	Social integration	Social support	Mastery	Self-esteem	Sense of coherence	Trust
Education	–	0.44**	0.32**	0.14**	0.14**	n.s.	n.s.	0.13**	n.s.	n.s.
Occupational status^1^	0.44**	–	0.26**	0.17**	0.13**	n.s.	0.07*	0.12**	n.s.	n.s.
Household income	0.32**	0.26**	–	0.27**	0.23**	0.08**	0.15**	0.19**	0.09**	0.15**
Self-rated economy	0.14**	0.17**	0.27**	–	0.14**	0.15**	0.25**	0.22**	0.23**	0.15**
Social integration^2^	0.14**	0.13**	0.23**	0.14**	–	0.38**	0.31**	0.35**	0.36**	0.19**
Social support^3^	n.s.	n.s.	0.08**	0.15**	0.38**	–	0.23**	0.42**	0.26**	0.12**
Mastery	n.s.	0.07*	0.15**	0.25**	0.31**	0.23**	–	0.68**	0.56**	0.14**
Self-esteem	0.13**	0.12**	0.19**	0.22**	0.35**	0.24**	0.68**	–	0.57**	0.13**
Sense of coherence	n.s.	n.s.	0.09**	0.23**	0.36**	0.26**	0.56**	0.57**	–	0.25**
Trust	n.s.	0.07*	0.15**	0.15**	0.19**	0.12**	0.14**	0.13**	0.25**	–

^1^ Including manual workers and non-manual employees

* p≤0.05

**p≤0.01

**Fig 1:**
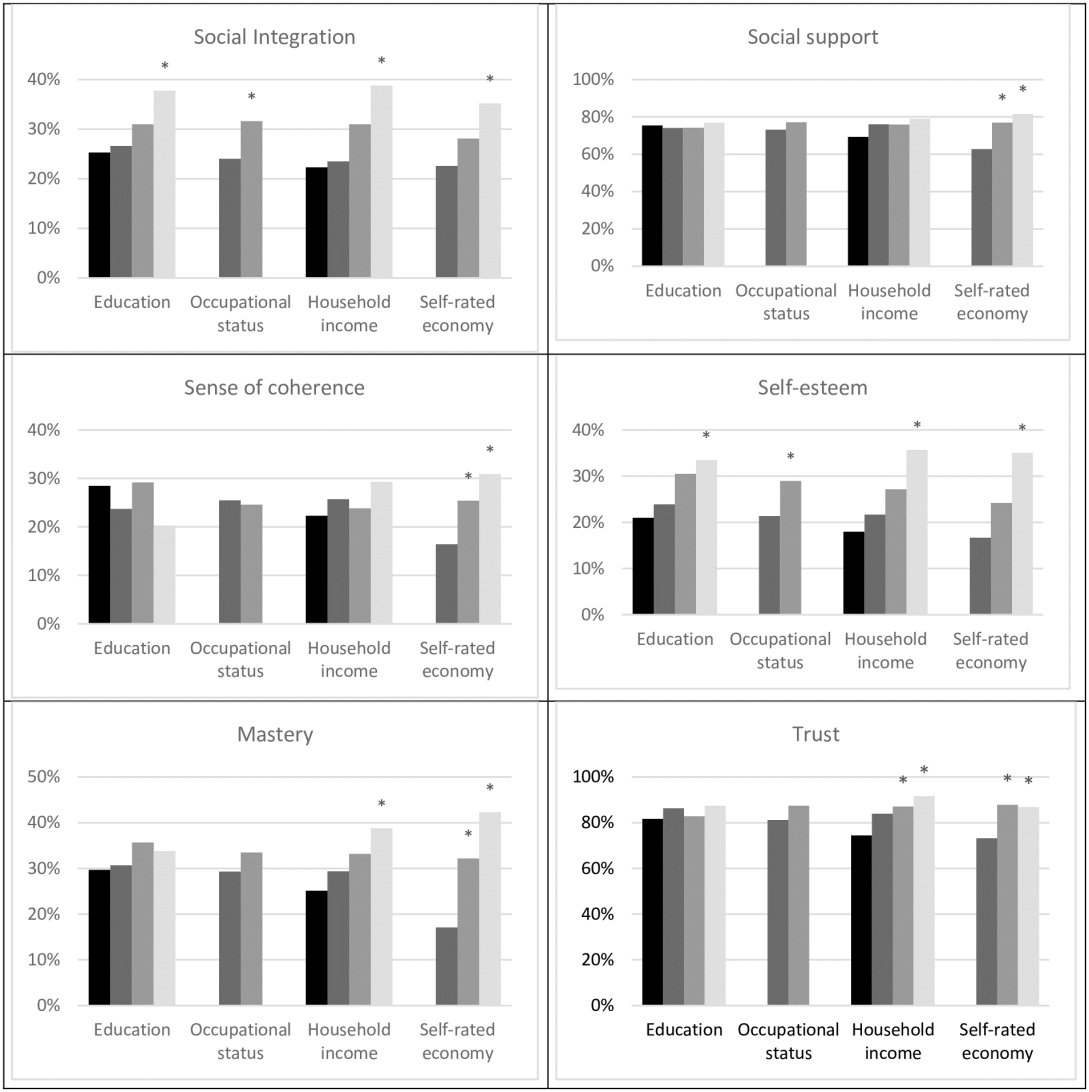
Prevalence of participants with high scale scores of psychosocial resources, stratified on SES measurements. *Statistically significant higher prevalence compared with the lowest group within each SES measure.

Fig 1 presents the relationships between SES and scale scores of psychosocial resources for each measure used. The bars in Fig 1 signify the prevalence of high resources presented across SES levels for each psychosocial measure and for four different SES indicators.

The most prominent findings were observed when measuring SES as self-rated economy, with significant differences for all six measures of psychosocial resources. Among the resources, social integration and self-esteem showed significant relationships with all four SES measures.

### Results after controlling for the effects of age and sex

Findings from the first step (adjusted for age and sex) of the logistic regression models are presented in Table 4. Household income and self-rated economy showed significant linear associations with all six measures of psychosocial resources. For occupational status, significant associations were seen for social integration, self-esteem and trust. For education, significant linear associations were seen for social integration and self-esteem.

**Table 4:** Logistic regression models of socioeconomic status and psychosocial resources, adjusted for age and sex.

		Social integration	Social support	Sense of coherence	Self-esteem	Mastery	Trust
Variable	Category	p* OR (95% C.I.)	p OR (95% C.I.)	p OR (95% C.I.)	p OR (95% C.I.)	p OR (95% C.I.)	p OR (95% C.I.)
**Education**		**0.002**	0.556	0.418	**0.001**	0.353	0.079
	Primary school	1.00	1.00	1.00	1.00	1.00	1.00
	Secondary school (2 years)	1.10 (0.76–1.59)	0.93 (0.64–1.36)	0.87 (0.60–1.27)	1.18 (0.80–1.75)	1.02 (0.71–1.46)	1.53 (0.98–2.41)
	Secondary school (3–4 years)	1.32 (0.84–2.09)	1.03 (0.63–1.66)	1.20 (0.76–1.90)	1.58 (0.99–2.53)	1.21 (0.78–1.88)	1.25 (0.72–2.17)
	University	1.83 (1.24–2.72)	1.11 (0.73–1.71)	0.74 (0.48–1.15)	1.87 (1.24–2.82)	1.15 (0.78–1.71)	1.76 (1.04–2.98)
**Occupational status**		**0.001**	0.402	0.325	**0.035**	0.074	**0.032**
	Manual workers	1.00	1.00	1.00	1.00	1.00	1.00
	Non-manual employees	1.48 (1.10–1.99)	1.22 (0.90–1.66)	0.98 (0.72–1.33)	1.50 (1.10–2.05)	1.22 (0.91–1.63)	1.61 (1.11–2.32)
	Self-employed and farmers	2.94 (1.61–5.33)	1.29 (0.65–2.57)	1.58 (0.848–2.99)	1.47 (0.77–2.83)	1.26 (0.68–2.33)	1.72 (0.70–4.22)
**Household income**		**<0.001**	**0.003**	**0.014**	**<0.001**	**0.006**	**<0.001**
	Q1	1.00	1.00	1.00	1.00	1.00	1.00
	Q2	1.06 (0.69–1.62)	1.44 (0.96–2.16)	1.20 (0.78–1.83)	1.25 (0.80–1.97)	1.22 (0.82–1.85)	1.83 (1.16–2.88)
	Q3	1.60 (1.05–2.43)	1.60 (1.06–2.43)	1.26 (0.81–1.95)	1.67 (1.07–2.60)	1.40 (0.93–2.10)	2.84 (1.74–4.66)
	Q4	2.30 (1.52–3.47)	1.94 (1.26–2.99)	1.77 (1.14–2.73)	2.48 (1.60–3.83)	1.77 (1.18–2.65)	4.77 (2.72–8.38)
**Self-rated economy**		**0.002**	**<0.001**	**0.001**	**<0.001**	**<0.001**	**<0.001**
	Quite bad	1.00	1.00	1.00	1.00	1.00	1.00
	Quite good	1.34 (0.92–1.96)	1.99 (1.40–2.83)	1.69 (1.11–2.56)	1.63 (1.07–2.48)	2.39 (1.59–3.60)	2.63 (1.74–3.96)
	Very good	1.88 (1.26–2.82)	2.62 (1.74–3.96)	2.18 (1.40–3.93)	2.80 (1.80–4.35)	3.76 (2.44–5.80)	2.37 (1.59–6.90)
**Country of birth**		**0.021**	0.965	0.269	0.493	0.061	**0.017**
	Nordic country	1.00	1.00	1.00	1.00	1.00	1.00
	Other	0.33 (0.13–0.85)	1.02 (0.49–2.11)	0.63 (0.27–1.44)	0.76 (0.34–1.68)	0.45 (0.20–1.04)	0.43 (0.21–0.86)
**Employment status**		**<0.001**	0.309	0.087	0.058	**0.003**	**<0.001**
	Working	1.00	1.00	1.00	1.00	1.00	1.00
	Unemployed	0.41 (0.24–0.70)	0.69 (0.43–1.11)	0.62 (0.37–1.06)	0.51 (0.29–0.90)	0.39 (0.23–0.68)	0.39 (0.22–0.68)
	Pensioner	0.40 (0.25–0.63)	0.87 (0.54–1.39)	0.65 (0.41–1.04)	0.79 (0.49–1.25)	0.75 (0.49–1.17)	0.22 (0.12–0.38)

* p for trend in education, household income, and self-rated income.

For the other variables, p is from the Wald statistic.

Looking at each of the six psychosocial resources, the relationship to SES measures varied depending on measure: social integration and self-esteem showed significant relationships with all four measures of SES, and trust was related to all SES measures except education. Social support, SOC and mastery were related to household income and self-rated economy.

Table 4 also presents relationships to psychosocial resources of possible confounders to SES; country of birth showed significant relationships to social integration and trust with lower scores for the group born outside Nordic counties. Being unemployed was significantly related to lower scores for social integration, self-esteem, mastery and trust. Pensioners scored lower on social integration and trust compared with working participants.

None of the interaction terms for SES and sex were statistically significant in any of the models in Table 4.

### Controlling for age, sex, country of birth and employment status

In the second step of the analyses, the regression models with SES and psychosocial resources were adjusted for two possible confounders: country of birth and employment status. All significant associations between SES and psychosocial resources described above remained (Table 5).

**Table 5:** Logistic regression models of socioeconomic status and psychosocial resources, adjusted for age, sex, country of birth and employment status.

		Social integration	Social support	Sense of coherence	Self-esteem	Mastery	Trust
Variable	Category	p* OR (95% C.I.)	p OR (95% C.I.)	p OR (95% C.I.)	p OR (95% C.I.)	p OR (95% C.I.)	p OR (95% C.I.)
**Education**		**0.006**	0.678	0.327	**0.002**	0.432	0.142
	Primary school	1.00	1.00	1.00	1.00	1.00	1.00
	Secondary school (2 years)	1.28 (0.73–1.54)	0.91 (0.63–1.33)	0.84 (0.58–1.23)	1.17 (0.79–1.73)	0.98 (0.69–1.41)	1.42 (0.89–2.25)
	Secondary school (3–4 years)	1.73 (0.80–2.05)	1.01 (0.62–1.65)	1.16 (0.73–1.85)	1.58 (0.98–2.54)	1.21 (0.77–1.89)	1.21 (0.69–2.15)
	University	1.13 (1.16–2.57)	1.07 (0.69–1.64)	0.71 (0.46–1.10)	1.82 (1.20–2.76)	1.11 (0.74–1.65)	1.62 (0.95–2.78)
**Occupational status**		**0.003**	0.510	0.396	0.066	0.567	0.072
	Manual workers	1.00	1.00	1.00	1.00	1.00	1.00
	Non-manual employees	1.44 (1.06–1.95)	1.19 (0.87–1.62)	0.97 (0.71–1.32)	1.45 (1.06–1.98)	1.17 (0.87–1.57)	1.54 (1.06–2.25)
	Self-employed and farmers	2.58 (1.41–4.74)	1.25 (0.63–2.50)	1.51 (0.79–2.87)	1.37 (0.71–2.63)	1.13 (0.60–2.10)	1.43 (0.58–3.54)
**Household income**		**<0.001**	**0.005**	**0.027**	**<0.001**	**0.037**	**<0.001**
	Q1	1.00	1.00	1.00	1.00	1.00	1.00
	Q2	0.96 (0.62–1.48)	1.45 (0.96–2.19)	1.17 (0.76–1.80)	1.24 (0.78–1.95)	1.19 (0.79–1.80)	1.73 (1.09–2.74)
	Q3	1.43 (0.93–2.19)	1.60 (1.05–2.44)	1.23 (0.79–1.93)	1.58 (1.00–2.49)	1.29 (0.85–1.96)	2.65 (1.58–4.42)
	Q4	1.99 (1.30–3.04)	1.91 (1.23–2.97)	1.68 (1.08–2.62)	2.30 (1.47–3.59)	1.56 (1.03–2.37)	3.95 (2.13–7.03)
**Self-rated economy**		**0.013**	**<0.001**	**0.002**	**<0.001**	**<0.001**	**0.004**
	Quite bad	1.00	1.00	1.00	1.00	1.00	1.00
	Quite good	1.18 (0.80–1.74)	1.96 (1.37–2.80)	1.57 (1.03–2.40)	1.57 (1.02–2.41)	2.30 (1.52–3.49)	2.26 (1.47–3.47)
	Very good	1.68 (1.12–2.54)	2.61 (1.72–3.97)	2.05 (1.31–3.20)	2.67 (1.70–4.19)	3.54 (2.27–5.51)	2.02 (1.25–3.26)

* p for trend in education, household income, and self-rated income. For the other variables, p is from the Wald statistic.

### Controlling for age, sex, country of birth, employment status and for other measures of SES

In the third step, the relationships above were tested on whether they sustained being mutually controlled for the other measures of SES (Table 6). For educational status, observed associations with social integration and self-esteem remained when controlling for occupational status, but were lost when controlling for household income and when controlling for both these measures (Table 6).

**Table 6:** Logistic regression models of socioeconomic status and psychosocial resources, adjusted for age, sex, country of birth, employment status and other measures of SES.

		Social integration	Social support	Sense of coherence	Self-esteem	Mastery	Trust
Variable	Category	p* OR (95% C.I.)	p OR (95% C.I.)	p OR (95% C.I.)	p OR (95% C.I.)	p OR (95% C.I.)	p OR (95% C.I.)
**Education**^1^		0.089	0.705	0.160	0.076	0.996	0.879
	Primary school	1.00	1.00	1.00	1.00	1.00	1.00
	Secondary school (2 years)	1.03 (0.70–1.53)	0.87 (0.59–1.29)	0.80 (0.54–1.19)	1.06 (0.71–1.61)	0.93 (0.64–1.36)	1.34 (0.83–2.18)
	Secondary school (3–4 years)	1.17 (0.70–1.97)	0.83 (0.49–1.42)	1.06 (0.63–1.77)	1.41 (0.83–2.38)	1.10 (0.67–1.80)	0.89 (0.47–1.68)
	University	1.52 (0.94–2.47)	0.91 (0.54–1.53)	0.61 (0.36–1.03)	1.50 (0.91–2.48)	0.94 (0.59–1.52)	1.09 (0.56–2.09)
**Occupational status**^2^		0.078	0.815	0.374	0.824	0.853	0.639
	Manual workers	1.00	1.00	1.00	1.00	1.00	1.00
	Non-manual employees	1.08 (0.74–1.56)	1.11 (0.77–1.62)	0.98 (0.67–1.43)	1.03 (0.70–1.52)	1.09 (0.76–1.55)	1.24 (0.78–1.97)
	Self-employed and farmers	2.06 (1.10–3.87)	1.17 (0.58–2.38)	1.56 (0.81–3.03)	1.24 (0.63–2.44)	1.15 (0.61–2.18)	1.23 (0.49–3.09)
**Household income**^3^		**0.009**	**0.010**	**0.011**	**0.003**	0.071	**<0.001**
	Q1	1.00	1.00	1.00	1.00	1.00	1.00
	Q2	1.02 (0.65–1.59)	1.51 (0.99–2.31)	1.14 (0.73–1.76)	1.23 (0.78–1.96)	1.13 (0.74–1.72)	1.86 (1.15–2.99)
	Q3	1.42 (0.92–2.20)	1.64 (1.06–2.53)	1.28 (0.82–2.02)	1.52 (0.96–2.41)	1.25 (0.82–1.91)	2.72 (1.61–4.59)
	Q4	1.71 (1.09–2.69)	1.91 (1.19–3.06)	1.86 (1.16–3.00)	2.04 (1.27–3.27)	1.49 (0.96–2.32)	4.01 (2.15–7.48)
**Self-rated economy**^4^		0.116	**<0.001**	**0.008**	**0.002**	**<0.001**	0.171
	Quite bad	1.00	1.00	1.00	1.00	1.00	1.00
	Quite good	1.08 (0.72–1.62)	1.75 (1.20–2.54)	1.55 (1.00–2.40)	1.46 (0.94–2.28)	2.17 (1.41–3.32)	1.87 (1.19–2.94)
	Very good	1.43 (0.92–2.22)	2.25 (1.43–3.53)	1.90 (1.18–3.06)	2.14 (1.33–3.45)	3.44 (2.17–5.48)	1.44 (0.85–2.43)

* p for trend in education, household income, and self-rated income.

For the other variables, p is from the Wald statistic.

^1^Adjusted for SES measures of occupational status and household income.

^2^Adjusted for SES measures of education and household income.

^3^Adjusted for SES measures of education and occupational status.

^4^Adjusted for SES measures of education, occupational status and household income.

For occupational status, the observed association to social integration remained when controlling exclusively for education, while associations to self-esteem and trust were lost. When controlling only for household income the associations to self-esteem and trust was lost, while associations to social integration remained. Finally, after controlling for both educational status and household income, associations with social integration, self-esteem and trust were all lost.

In the models for household income, the association with mastery was lost after controlling for education and occupation, both separately and together. In contrast, significant relationships to all other five psychosocial resources remained after controlling for education and occupational status.

In the models for self-rated economy all measures of psychosocial resources, except social integration and trust, remained statistically significant after controlling for education, occupational status and household income. Regarding both social integration and trust, the association remained after controlling for education and occupation exclusively, but was lost after controlling for household income, both when controlling separately and in combination with education and occupation.

### Differences in age, sex, country of birth and employment status

The models in Table 6 also revealed some significant differences regarding age, sex, and employment status; although not shown in the table, they may be of interest to mention briefly. After controlling for the effects of other SES measures, scores of social integration, SOC and trust increased significantly with higher age, men reported lower scores of social support and trust compared with women, and pensioners reported lower scores on social integration and trust compared with people in work.

## Discussion

### Main findings

In a normal middle-aged Swedish population, we found that the relationship between SES and psychosocial resources differed depending on the measures used. The strength of the observed relationships was contingent on the choice of measure for both SES and psychosocial resources. The results indicated that income-related SES measures (self-rated economy and household income) exhibited the most consistent relationship with psychosocial resources. Among the latter, social integration and self-esteem showed a significant relationship with all four SES measures. Our findings therefore do suggest that the choice of measure matters when investigating SES as well as psychosocial resources.

#### Findings regarding choice of SES measure

Our findings that different SES indicators, although correlated, had different relationships to the six measures of psychosocial resources are in line with previous research where studies have shown that relationships to health outcome for different SES measures differ depending on the measure of health. Our data therefore support, as suggested by other authors, that these different measures, i.e. education, occupation and income, capture different facets of SES and therefore cannot be used interchangeably as indicators of a hypothetical latent social dimension [25].

We found that self-rated economy and household income showed a more consistent relationship with psychosocial resources compared with occupation and education. The fact that self-rated economy and (self-rated) psychosocial resources are highly correlated might not be so surprising because they both signify subjective evaluations. However, household income also showed strong and independent relationships to all six investigated resources. This is consistent with previous research showing a link between income-related SES measures and psychosocial resources e.g. self-esteem [36] and mastery [37]. Possible explanations for these findings are that the effect of income translates into material or immaterial resources for health, such as better housing, clothing, food, and resources for mastering stressful and demanding situations and to control one’s circumstances [25]. In this normal population in Sweden, with a long tradition of welfare regime, it is notable however, that income had the most prominent relationships with psychological resources.

Educational and occupational status were the SES measures with the weakest relationship with psychosocial resources. Only two of the six measures (social integration and self-esteem) showed significant relationships with education and three (social integration, self-esteem and trust) showed significant relationship with occupation. Moreover, after controlling for other SES measures, these observed relationships were lost. Notably, this was in both cases mainly an effect of income. Previous research have shown that income mediate the effect of occupation on increased risk of myocardial infarction [38] and also the effect of education on self-rated heath [39]. Education is the most commonly used measure of SES and it provides trustworthy data in terms of reliant self-reports and exhibits limited change over time. Education can be seen as an indicator of childhood social environment but also differentiate between level of awareness in adulthood e.g. access to knowledge, competency, increased health literacy and better access to the labour market. However, information on educational level, typically measured retrospectively, may offer information on an individual’s current life situation which is different from measures of income and occupational status [6,26]. This could be particularly true for studies using a middle- or high-aged population. Occupation as a measure of SES is thought to reflect several aspects associated with social status such as prestige, income and educational achievement, and has been seen as a major structural link between education and income. Occupational status can give information on work environment and work conditions of a social group including both the physical and psychosocial work environment e.g. effort/reward and job demand/control balance [26]. A limitation of using occupation as an indicator of SES is that it generally does not include individuals outside of the workforce e.g. the unemployed [26]. However, our data included data on lifetime occupation status for all participants. In studies of SES differences in health, measures of social integration, social support and self-esteem have partly explained educational differences in health, whereas studies on occupation mainly have examined work conditions and not resources in general [6].

#### Findings regarding choice of psychosocial resource measure

We also found that individual psychosocial resources were related to each SES dimension differently. Only self-esteem and social integration were consistently related to all four SES measures. The findings on self-esteem support previous research showing a robust relationship with SES. In contrast to our results, a meta-analysis including studies from many countries showed a stronger relationship to self-esteem for education and occupation than for income [36]. These inconsistencies could be explained by cultural factors considering that the meta-analysis included multiple cultural contexts. Indeed, this review included hundreds of samples from e.g. Europe, Africa and Asia and age groups throughout the life span. The self-esteem and SES relationship was found to vary across the lifespan and between cultural contexts, e.g. the relationship was stronger among Asian and Asian American groups compared to Hispanics.

Moreover, our findings add to previous research on a relationship between social integration and SES by showing consistent links between social integration and all four indicators of SES. Measures of social relations, in terms of perceived instrumental support have demonstrated a mediating effect on the association between SES and self-rated health. Significant reductions have been found for education, occupation and household income, with the strongest effect for income [40,41].

In contrast to findings for self-esteem and social integration, the relationship between the other four resources (trust, social support, mastery and SOC) and SES differed depending on the SES measure. Trust, measured with a single item, was significantly related to all SES measures except education. Research has shown that high levels of trust in neighborhood (high social capital), among Canadian youths reduced SES differences in health [42]. However, to our knowledge, there is limited research looking at the relationship of individual measures of trust and SES, and our findings suggest that this relationship is worth further investigation. Regarding social support, we found independent relationships to household income and self-rated economy. Previous research has shown that social support (emotional support), partially explained SES inequalities in self-rated health. This was shown for SES in terms of education, occupation and household income, and consistent with our findings, the effects were shown to be the strongest for income [40,41]. Regarding mastery and SOC, our findings showed relationships with both household income and self-rated economy. In an earlier study of 50-year-old men, relationships between mastery and SES were found for education, occupation and income [43]. Regarding SOC, research is limited and the findings are incongruent; SOC has been reported to be higher in manual workers and self-employed groups compared with non-manual workers [44], but also the inverse relationships have been reported [45].

The measures were moderately to strongly correlated; the highest correlation, between mastery and self-esteem, was 0.68 and thus the common variance was 46%. Although research on psychosocial resources shows that constructs are interrelated, our analysis do not support the suggestion of a common core [46,47]. Our findings suggest that they do represent different constructs and should be used as such.

### Methodological considerations

A major strength of this study is that the study population is well characterized and a comprehensive set of psychosocial instruments and measures of SES are used, making the proposed analyses possible. Another strength is that it includes a random sample from the normal population in Sweden that was representative of the population in terms of education, employment rate and immigrant status, and the range of scale scores in our study covered the theoretical possible range for the instruments used. Furthermore, occupational status was measured by life career (occupation held during major part of life). Thus, the data could be stratified for occupational status on all participants and also on employment status (working, unemployed and pensioner). Another strength is that we controlled for potential confounders in several steps; first for age and sex, second for the effect of employment status and country of birth, and finally for all other SES measures. The results discussed in the article are based on the second step. This could make our conclusions more conservative compared with previous studies. However, the results were comparable in these two analyses. We also adjusted for the effects of other SES measures to identify the independent effect of each SES measure.

A possible limitation is the response rate of 62.5%. Even though this is an expected response rate, it still impose a potential selection bias. We know that a higher proportion of non-responders can be found among low SES groups. However, such a selection bias would result in a conservative interpretation of a potential effect.

The statistical power of the study is a potential limitation. However, our findings show a distinct pattern with little indication that our conclusions are an effect of low power. In these fundamental analyses, sex was included as a possible confounder. In coming studies, we will also stratify for sex because previous studies have shown gender differences in psychosocial resources [48]. Also, with a larger data set it would be valuable to analyse occupational status on a more detailed level. Although Swedish national databases, are known to have high quality and validity, a possible limitation is that cohabitation is not taken into account when calculating disposable household income. That is, individuals who live together without being married (or having children together) are registered as single, so only individual disposable income is available for those persons. Also, number of persons living together, e.g. children, was also not taken into account. Thus, household income as an SES indicator could be underestimated. However, in Sweden, most couples who live together are married (70%), suggesting that most of the household income data in this study were representative of an individual’s financial situation [49]. If anything, this potential bias would lead to underestimation of differences. We also included a measure of self-rated economy, which could imply challenges due to its subjective nature. However, register-data on economy is not always available. Therefore, we wanted to investigate this subjective measure of economic status: how it related to psychosocial resources and how it potentially differed in this respect to register data. Self-rated economy is also interesting per se as it represents the individual’s perception of his/her financial situation, which can also be influenced by the comparison to the situation of others. Finally, the study used a cross-sectional design and we can therefore not draw any conclusions regarding the direction of the relationships. However, our findings offer valuable knowledge on the interrelationships between determinants of health inequalities. These thorough fundamental analyses have often been neglected in previous research looking at health inequalities [24].

## Conclusions

We found, in this population-based study of a middle-aged population in Sweden, that the associations between SES and psychosocial resources did differ depending on the measures used. Income-related SES measures (self-rated economy and household income) exhibited the most consistent relationship with psychosocial resources. Among the latter, social integration and self-esteem showed a significant relationship with all four SES measures. The findings illustrate the importance of the choice of measure when investigating SES as well as psychosocial resources.

## Supporting information

S1 File Data file. Data underlying the findings.
(SAV)
